# Low-Speed Platelet-Rich Fibrin Membrane in Conjunction With Demineralized Freeze-Dried Bone Allograft (DFDBA) Compared to Collagen Membrane With DFDBA in Noncontained Intraosseous Defects of Stage III Periodontitis: A Randomized Controlled Clinical Trial

**DOI:** 10.1155/ijod/6393105

**Published:** 2025-02-23

**Authors:** Najeeb Almoliky, Manal Hosny, Weam Elbattawy, Karim Fawzy El-Sayed

**Affiliations:** ^1^Oral Medicine and Periodontology Department, Faculty of Dentistry, Cairo University, Cairo, Egypt; ^2^Clinic for Conservative Dentistry and Periodontology, School of Dental Medicine, Christian Albrechts University, Kiel, Germany; ^3^Stem Cells and Tissue Engineering Research Unit, Faculty of Dentistry, Cairo University, Cairo, Egypt

**Keywords:** collagen, intraosseous, membrane, periodontitis, platelet-rich fibrin, regeneration

## Abstract

**Aim:** Noncontained (1- or combined 1- to 2-wall) periodontal intraosseous defects represent challenging clinical situations with unpredictable surgical therapeutic outcomes. This randomized clinical trial assessed demineralized freeze-dried bone allograft (DFDBA) with low speed-platelet-rich fibrin (PRF) membrane compared to DFDBA with collagen membrane (CM) in the surgical periodontal therapy of noncontained intraosseous defects of stage III periodontitis patients.

**Methodology:** Twenty-two stage III periodontitis patients with noncontained intraosseous defects measuring ≥3 mm and clinical attachment loss ≥5 mm were randomly allocated into two groups: test group (low-speed PRF membrane + DFDBA) and control group (CM + DFDBA), with 11 participants per group. Clinical and radiographic assessments were conducted at baseline, 3, 6, 9, and 12 months for clinical attachment level (CAL; primary outcome), gingival recession depth (GRD), probing depth (PD), full mouth bleeding score (FMBS) and full mouth plaque score (FMPS), radiographic bone fill and radiographic linear defect depth (RLDD; all secondary outcomes).

**Results:** The mean (±SD) CAL-gain for the test group was 2.45 (±1.51), 2.91 (±1.70), 2.91 (±1.87), and 2.82 (±1.83) mm, while for the control group 2.82 (±1.25), 3.27 (±1.27), 3.00 (±1.41), and 2.64 (±1.50) mm at 3, 6, 9, and 12 months, respectively, with no significant intergroup differences (*p* > 0.05). Despite the absence of significant intergroup differences, both groups demonstrated significant intragroup improvement in CAL- and PD-gain, and RLDD-reduction at 3, 6, 9, and 12 months as well as RLDD improvement at 12 months (*p* < 0.05).

**Conclusion:** PRF membranes, in conjunction with DFDBA, show significant improvement of periodontal clinical and radiographic parameters, comparable to CMs with DFDBA.

**Trial Registration:** ClinicalTrials.gov identifier: NCT03922503

## 1. Introduction

Periodontitis is an inflammatory multifactorial damaging ailment of periodontal elements correlated with microbiological dysbiosis [1, 2]. The primary goal of periodontal therapy remains to be the arrest the progressive destructive inflammatory disease process, ultimately reducing the risk of tooth loss and possibly restoring the tissues lost due to periodontitis [3]. Still, the ideal goal of regenerative therapy is the reconstruction of the destructed periodontium (periodontal ligament, gingiva, cementum, and alveolar bone) [4], thus improving long-term tooth prognosis [5]. Regenerative periodontal therapy, often involving a combination of nonsurgical and surgical periodontal therapeutic approaches, is currently recommended for the treatment of intraosseous defects [6, 7]. Especially the regeneration of noncontained intraosseous defects is considered challenging, with approaches involving the combination of barrier membranes and bone grafts commonly advocated [6].

Platelet-rich fibrin (PRF) is an autologous matrix rich in fibrin, platelet, cytokines, as IL-1*β*, -4, and -6, and growth factors, including fibroblast growth factor-*β* (FGF-*β*), insulin-like growth factor (IGF), platelet-derived growth factor (PDGF), transforming growth factor-*β* (TGF-*β*), and vascular endothelial growth factor (VEGF) [8–12]. The introduction of the low-speed centrifugation concept lead to enhanced PRF formulations with increased platelets, growth factors, and leucocytes within their matrices [13] and higher cytokine/growth factors emission properties in contrast to earlier PRF preparation protocols [14], with positive impacts on inflammation, vascularization and periodontal wound healing events [15–19]. A number of studies demonstrated that the amalgamation of PRF with bone grafts could result in significantly greater improvement in clinical attachment level (CAL), gingival recession, probing depth (PD), and defect fill compared to bone grafts alone [20–24].

Demineralized freeze-dried bone allograft (DFDBA), harboring bone morphogenetic proteins (BMPs 2, 4, and 7) [25], has been widely used, usually in combination with collagen membranes (CMs), in periodontal intraosseous defect therapy with multiple reported advantages [26]. The aim of the present randomized controlled trial was to compare for the first time the clinical and radiographic outcomes after using low-speed PRF membrane versus CM in combination with DFDBA in surgical therapy of noncontained periodontal intraosseous defects of stage III periodontitis patients. The null hypothesis defined was that there would be no difference in CAL gain between the test and control groups.

## 2. Material and Methods

### 2.1. Study Registration and Design

This study employed a randomized, parallel arms, double-blind, controlled trial design with a 1:1 allocation ratio. The objective was to evaluate the radiographic and clinical outcomes of using a low-speed PRF membrane with DFDBA (test group) versus CM with DFDBA (control group) in the surgical treatment of noncontained intraosseous defects (one wall or combined 1- to 2-wall defects) in patients with stage III periodontitis. The study protocol and consent forms were authorized by the Ethical Committee of the Faculty of Dentistry, Cairo University, Egypt, in April 2019 (approval: 19|04|03). The trial adhered to the EQUATOR guidelines and followed the ethical principles outlined in the Declaration of Helsinki for medical research with humans (revised in Fortaleza 2013), and the CONSORT checklist was employed for reporting this randomized controlled trial outcomes [27].

### 2.2. Population

**Figure 1:**
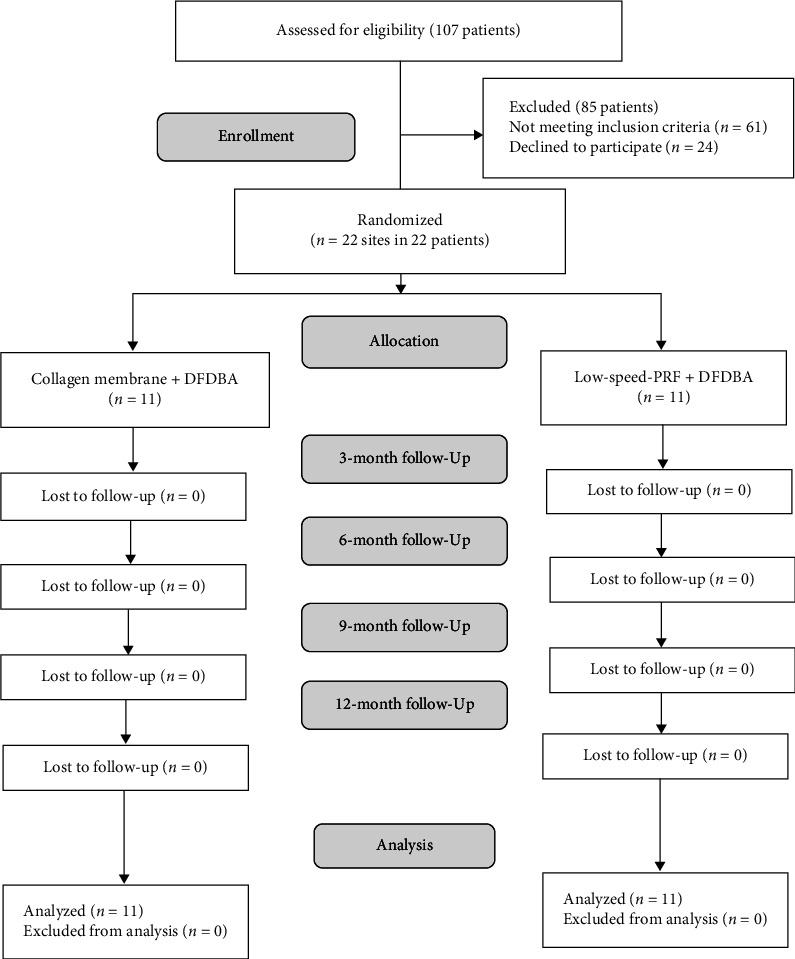
Flow diagram of patient recruitment and follow-up.

Patient recruitment, treatment, and follow-up were conducted at the Faculty of Dentistry, Cairo University, Egypt, from October 2019 to September 2021. The recruitment process involved screening of patients, personal referrals, and poster announcements. In total, 107 patients were initially assessed for eligibility, of which 85 were excluded for either not meeting the inclusion criteria (self-reported smoking, pregnancy, and diabetes) or refusing to participate in the study. Eventually, 22 patients with 22 defects were enrolled in the study, as demonstrated in Figure 1. Patients included in this study were ≥18 years old, diagnosed with stage III periodontitis with one wall or combined 1- to 2-wall intraosseous defects (diagnosed via periapical radiographs and to be confirmed after flap reflection), did not have preceding surgeries at the treatment site, with PD ≥ 6 mm and CAL ≥ 5 mm and intraosseous defect depth ≥3 mm radiographically, persisting 6–8 weeks after initial nonsurgical periodontal therapy [6], and full mouth bleeding score (FMBS) [28] and full mouth plaque score (FMPS) [29] less than 20% [30]. The study excluded patients with mobile teeth, intraosseous defects that extended to furcations, medical conditions known to disturb periodontal healing (such as diabetes or hyperthyroidism), smokers, and former smokers [31]. Additionally, patients undergoing active orthodontic treatment, those with a history of chemotherapy, radiotherapy, or bisphosphonate use, as well as pregnant or lactating ladies, were excluded from the trial [32, 33].

### 2.3. Sample Size

A mean CAL-difference of 0.5 mm was considered as the minimally important difference with a standard deviation of 0.36 mm [34]. With a power (*β*) of 80% and *α* of 5% using a two-tailed *t*-test, it was determined that a minimum of nine defects would be necessitated per group in the study. To make-up for potential dropouts, the sample was increased to 11 defects per group. Calculation of the sample size was carried out using the PS software (v 3.1.2, Vanderbilt University, Tennessee, USA).

### 2.4. Randomization and Blinding

A randomized sequence was produced (1:1 allocation) using a computer-generated randomization list via www.random.org. Allocation concealment was masked within indistinguishable, serialized, and opaque closed envelopes (Manal Hosny). All patients were uniformly prepared for their surgery and operated on by the same periodontist (Najeeb Almoliky). The assignment of patients to either the test group (low-speed PRF + DFDBA) or the control group (CM + DFDBA) was carried out by the study coordinator (Karim Fawzy El-Sayed) after flap reflection and completing the defect debridement process where the defect morphology was confirmed (one wall or combined 1- to 2-wall noncontained intraosseous defects). Defects that did not match the inclusion criteria received treatment without breaking the concealment for them and were not included in the study.

Owing to the design of the study, it was not possible to blind the patients or the operator (Najeeb Almoliky). However, the outcome assessors and the statistician remained blinded throughout the study.

### 2.5. Parameters

The CAL-gain was defined as the study's primary outcome, while gingival recession depth (GRD), PD, FMBS, FMPS, radiographic bone fill, and radiographic linear defect depth (RLDD) were all defined as secondary outcomes.

UNC-15 periodontal probes and prefabricated custom stents were employed for standardization at baseline, 3, 6, 9, and 12 months postoperatively to measure the CAL-gain (primary outcome), CAL, PD, and GRD, as well as FMPS and FMBS at 12 months (all secondary outcomes) according to the criteria described before. Furthermore, radiographic analysis, using standardized periapical radiographs, was conducted using the paralleling method and E-speed films (YES!Star, Zhengzhou Smile Dental Equipment Co., Ltd, Zhengzhou, China), as depicted before to record RLDD at baseline, 6, 9, and 12 months, bone fill and percentages of bone fill as a proportion of the baseline RLDD [22]. Anatomical structures such as alveolar crest (AC) and defect base (DB) were identified to measure the defect depth as RLDD, which was measured from the AC to the DB as previously reported [33].

### 2.6. Calibration

All parameters were recorded by two blinded periodontists (Weam Elbattawy and Manal Hosny). Preceding the trial, both examiners underwent an intraexaminer calibration process. This involved comparing two measurements of the same examiners on three individuals who were not part of the study, with a 1-week interval between the measurements. The intraexaminer correlation scores obtained for all clinical measurements were 0.85, while for radiographic outcomes, the scores were 0.82.

### 2.7. Interventions

#### 2.7.1. Presurgical Phase

**Figure 2:**
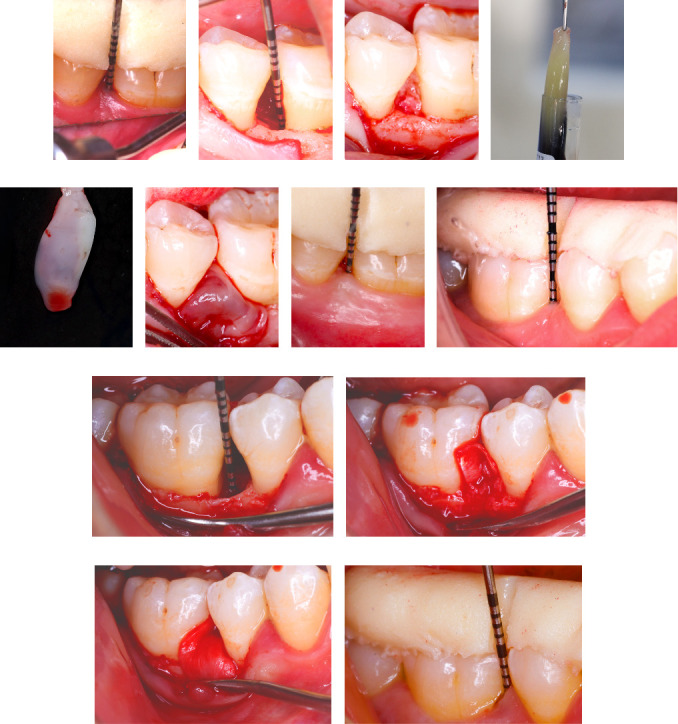
Clinical steps in representative cases of the test (a–g) and control (h–l) groups. Test group using low-speed PRF/DFDBA and control group using collagen membrane/DFDBA. Test group: (a) baseline measurements taken using a prefabricated stent, (b) intraosseous defect at the mesial site of the lower left first molar, (c) application of DFDBA in the defect, (d) low-speed PRF preparation, (e) low-speed PRF as a membrane, (f) low-speed PRF membrane covering the defect, and (g) final clinical measurements 12 months postoperatively. Control group: (h) baseline measurements taken using a prefabricated stent, (i) intraosseous defect at the mesial site of lower right first molar, (j) DFDBA and collagen membrane at the defect, (k) adaptation of the collagen membrane to the defect, and (l) final clinical measurements 12 months postoperatively. DFDBA, demineralized freeze-dried bone allograft; PRF, platelets-rich fibrin.

Prior to obtaining the informed consents, all eligible participants were provided with a detailed explanation of the study procedures and timeline. Phase I therapy commenced, which involved nonsurgical treatment consisting of ultrasonic supragingival and subgingival debridement. Additionally, oral hygiene instructions were provided, including toothbrushing and twice-daily use of a 0.12% chlorhexidine HCL (Hexitol, ADCO Pharma Co, Cairo, Egypt) for a duration of 2 weeks [6]. Following a period of 6–8 weeks, a re-evaluation was conducted to determine the need for surgical intervention based on the presence of interproximal defects with PD ≥6 mm, CAL ≥5 mm, and intraosseous defect depth ≥3 mm radiographically [35]. To ensure consistent and standardized clinical measurements throughout the study acrylic stents harboring interproximal guiding grooves were individually constructed for each patient on study models (Figure 2). These custom-made stents served as guides for accurate measurements. Furthermore, bite registration blocks were constructed to assist in aligning the film holder, thereby standardizing the periapical radiographs using the paralleling technique.

#### 2.7.2. Surgical Phase

All surgical procedures were conducted by a single periodontist (Najeeb Almoliky). Local anesthesia was administered using 2% mepivacaine HCl with levonordefrin 0.005% (Alexandria Co. for Pharmaceuticals, Alexandria, Egypt). Incisions were done using a #15c blade intrasulcular. Full-thickness mucoperiosteal flaps were elevated both lingually/palatally and buccally around the affected tooth. Ultrasonic scalers (Woodpecker Medical Instrument Co., Guilin, China) and mini-/after-five Gracey curettes (Hu-Friedy, Chicago, USA) were used to debride the surgical site and remove any granulation tissue. The morphology of the defect was visually examined and documented to confirm the presence of noncontained (1- or combined 1- to 2-wall) intraosseous defects.

To produce the low-speed PRF, 10 mL of fresh blood was collected via venipuncture from the forearm of the patient and placed in a sterile glass vacuum tube (16 × 100 mm, 10 mL, Voma Med, Chongqing, China). The blood was processed according to previously reported methods [14, 36]. A digital tabletop centrifuge (VE-4000, Velab, TX, USA) was employed for the process, utilizing a rotation angle of 45° and a maximum radius of 10.6 cm. The centrifuge revoluted at 1300 RPM, resulting in a maximum relative centrifugal force of 200 *g*, and the duration of centrifugation was set at 8 min. Subsequently, the low-speed PRF clot was taken out from the tube and compressed using a sterile PRF box (Manson, Bejing, China), resulting in the formation of a low-speed PRF membrane.

After the surgical site was prepared, DFDBA granules were hydrated with saline and gently placed into the defect until they reached the level of the AC. The defect was then covered with the low-speed PRF membrane. In the control group, DFDBA (AlloOss; demineralized cortical particulates, ACE Surgical Supply Co., Brockton, MA, USA) was packed into the intraosseous defect and a bioresorbable CM (Evolution Fine OsteoBiol, Tecnoss Dental S.R.L., Torino, Italy) was trimmed and carefully adapted to cover the entire defect covering additionally 2–3 mm of the surrounding alveolar bone. The flap in both groups was passively repositioned and secured using 5-0 silk vertical mattress sutures (ASSUT Medical, Pully-Lausanne, Switzerland).

#### 2.7.3. Postoperative Phase

Following the surgical procedure, participants were instructed to take 1 g of Augmentin (GlaxoSmithKline, Worthing, England) containing 875 mg amoxicillin +125 mg clavulanate potassium twice daily for a duration of 7 days. Additionally, they were advised to take Ibuprofen 600 mg (Brufen, Kahira Pharma Co., Cairo, Egypt) twice daily for 3 days to manage pain and inflammation [37]. The participants were provided with instructions to refrain from brushing or causing any trauma to the surgical site. Additionally, they were advised to rinse their mouth twice daily for a period of 2 weeks using a 0.12% Chlorhexidine HCl mouthwash (Hexitol, ADCO Pharma, Cairo, Egypt) [37. Sutures were taken out 14 days following the surgery, and the patients were advised to brush carefully with a soft toothbrush. A weekly follow-up schedule was established during the first month, which was later repeated at 3, 6, 9, and 12 months after the surgery. These follow-up appointments served to assess visually healing of the surgical site and post-operative adverse effects after regenerative surgical procedures as pain, wound dehiscence, membrane exposure, abscess formation, and papilla necrosis [38], in addition to evaluation of the study outcomes and verification of adherence to proper oral hygiene practices.

### 2.8. Statistical Analysis

Descriptive statistics such as mean ± standard deviation or median with interquartile range were utilized to present numerical data. Categorical data were presented as both the number (*n*) and percentage (%), and the chi-square test was used for analysis. The normality of the data was assessed using the Kolmogorov–Smirnov and Shapiro–Wilk tests. For normally distributed data, intergroup comparisons were conducted using the independent *t*-test, while intragroup comparisons between time points were performed employing repeated-measures-ANOVA. Nonnormally distributed data were analyzed using the Mann–Whitney *U* test for intergroup comparisons and the Friedman test, followed by the post hoc Wilcoxon signed-rank test for intragroup comparisons. For multiple comparisons, Bonferroni correction was applied. A linear regression model was employed with CAL after 12 months as the dependent variable, exploring independent variables such as treatment (group), defect morphology, RLDD, radiographic defect angle (RDA), FMBS, and FMPS at baseline. All statistical tests were two-tailed, and a significance level of *p* < 0.05 was considered statistically significant (SPSS, version 26, IBM, New York, USA).

## 3. Results

### 3.1. Baseline Data

This parallel-group randomized trial involved 22 participants (14 females and 8 males) diagnosed for stage III periodontitis. A total of 22 noncontained intraosseous defects were included in the study, with no dropouts. These defects in each patient were randomly allocated to either the low-speed PRF + DFDBA group (*n* = 11, test group) or the CM + DFDBA group (*n* = 11, control group). The test group consisted of five females and six males, while the control group consisted of nine females and two males. The mean age of participants in the test group was 32.64 ± 6.82 years, while in the control group, it was 34.55 ± 8.31 years. The low-speed PRF + DFDBA group involved five anterior and six posterior teeth with 36.4% one-wall and 63.6% combined one–two wall defects, while the CM + DFDBA group had three anterior and eight posterior teeth with 45.5% 1-wall and 54.5% combined 1- to 2-wall defects. Baseline characteristics are represented in Table 1.

### 3.2. Clinical Parameters

Healing at all sites was without complications, where both groups demonstrated statistically significant CAL gain (*p* < 0.001) and PD reduction at 3, 6, 9, and 12 months postoperatively (*p* < 0.001, repeated measure ANOVA), with no significant intergroup differences observed (*p* > 0.05, independent *t*-test) while no significant intra- or intergroup differences regarding GRD were notable at any time point (*p* > 0.05; Table 2).

The low-speed PRF + DFDBA group and the CM + DFDBA group both exhibited similar findings in plaque scores (FMPS) and significant improvements in bleeding on probing scores (FMBS) at 12 months, with no significant intergroup differences (*p* ≥ 0.05, independent *t*-test; Table 2).

Regarding the clinical endpoint for periodontal therapy, all treated sites in the two groups showed PD of 4 mm or less. In both groups, there was a statistically significant decrease in PD after 12 months (*p* < 0.001), yet at baseline as well as after 12 months, there was no significant difference between the two groups (*p*=1, *p*=0.666, respectively, Table 3).

### 3.3. Radiographic Parameters

RLDD was significantly reduced within each group (*p* < 0.05, repeated measure ANOVA) over time, with no significant intergroup differences observed at any time point (*p* > 0.05, independent *t*-test). Both test and control groups resulted in significant millimeter and percentage radiographic bone fill compared to baseline but with no statistically significant intergroup differences (*p* > 0.05, Table 4).

### 3.4. Stepwise Linear Regression Analysis

A stepwise linear regression model was constructed using CAL after 12 months as the dependent variable. Treatment (group), defect morphology, RLDD, RDA, FMBS, and FMPS at baseline were the independent variables. The model was adjusted for age and gender. Results of the regression model showed that site was the only statistically significant predictor for CAL after 12 months. Anterior teeth sites showed statistically significantly higher mean CAL after 12 months than posterior teeth sites (*p* < 0.001, Table 5).

## 4. Discussion

Noncontained periodontal intraosseous defects represent challenging defect morphologies, affecting the prognosis of surgical periodontal therapeutic approaches [39, 40]. In the context of a guided tissue regeneration (GTR) approach, with the triad of compartmentalization, space maintenance, and clot stabilization, for undisturbed tissue maturation, remaining essential [41], a combination of periodontal regenerative biologics, grafts, and barriers has been advocated in the treatment of noncontained intraosseous defects [7, 42–44]. CM membranes have been employed in the current study as the “gold standard membrane” against which the effect of the low-speed PRF membrane would be tested. Similar to earlier findings [45, 46], the noncontained nature of the 1-wall and combined 1- to 2-wall intraosseous defects included in the present study necessitated the application of a grafting material, namely DFDBA, to support the barrier membrane, preventing any possible collapse into the defect or jeopardization of its space provision function essential for biological periodontal healing/regeneration events [26, 47]. DFDBA, in-turn, harbors several growth factors, particularly TGF-*β*1, VEGF, FGF-a, IGF-I, and BMPs 2, 4, and 7, and is implied to possess the ability for a slow release of BMPs, promoting periodontal wound healing and bone formation, with significant CAL-gain, PD-reduction, and bone fill [48, 49]. Thus, the current randomized trial compared low-speed PRF to collagen as barrier membranes in conjunction with DFDBA in the guided tissue regenerative approach of noncontained intraosseous periodontal defects of stage III periodontitis patients over a 12-month follow-up.

Earlier results demonstrated that low-speed PRFs produced via fixed-angle centrifugation techniques were reproducible with comparable quality, regardless of the centrifugation machine, when using equal centrifugal force and speed [50]. The current trial prepared the low-speed PRF membrane according to earlier described protocols [15, 51]. In accordance with current evidence supporting the application of PRF in periodontal intraosseous defects for enhanced CAL-gain, PD-reduction, radiographic bone fill, and favorable soft tissue outcomes [17, 52], the current study aimed to produce an autologous biological occlusive barrier enriched in growth factors for periodontal healing/regeneration [53], including PDGF, TGF-*β*, IGF, and FGF [54, 55]. Earlier results applying PRF and DFDBA together demonstrated significantly greater improvement in PD, CAL, gingival recession, and bone fill [20, 22]. Used as an occlusive barrier in a GTR approach and prepared using the low-speed preparation protocol, in the present trial, low-speed PRF + DFDBA demonstrated significant CAL-gain, PD-reduction, and radiographic defect fill over the study period comparable to CM + DFDBA in noncontained intraosseous defects.

Although it would be expected that due to the considerably lower degradation time of the CM in contrast to the 2–4-week degradation duration of the PRF membrane, the control group would show better clinical outcomes, both groups demonstrated comparable results. This could be partly explained by the fact that both groups employed DFDBA, which itself, as depicted above, harbors an array of essential growth factors, by themselves adequate to bring about remarkable periodontal healing/regeneration [48, 49], irrespective of the type of barrier membrane employed. Second, during its degradation process, it would be expected that the PRF membrane would, in contrast to the CM barrier, further provide a fibrin network, essential for any healing process, and enrich the healing wound area by the numerous growth factors outlined above, stimulating its immediate periodontal environment for a subsequent period of time during its healing and remodeling [56]. Especially the PRF membrane prepared through the low-speed centrifugation model would upsurge the concentration of granulocytes with more evenly distributed macrophages, exert antibacterial effects [57], and gradually release higher amounts of growth/differentiation factors over time [14]. These biological attributes could provide an additional synergistic potential to DFDBA, further promoting its periodontal repair/regeneration activity. PRF is further suggested to activate phosphorylated extracellular signal-regulated osteoprotegerin, alkaline phosphatase, and protein kinases, in addition to providing antimicrobial effects [58] and reducing the detrimental effects of the inflammatory processes [59], all biological effects not provided by the CM. Combined this could explain the absence observed of a clinical difference between test and control groups despite the considerably lower resorption rate of the CM.

It should also be emphasized that the clinical and radiographic efficacy of PRF in intraosseous defects was evident in the literature when compared to OFD [60–62] with significant impact and better clinical outcomes when additionally amalgamated with bone grafting materials or when compared to grafting materials alone [63, 64]. Thus, PRF, although linked to a higher cost than OFD debridement (in terms of blood withdrawal devices, test-tubes and centrifugation machines), offers in addition to its autologous nature, added clinical benefits with relative easiness and safety as a biological reparative/regenerative adjunctive intervention [65].

Despite the significant CAL gain and PD reduction values observed in both groups of this clinical study, it is important to highlight the value of reaching a valuable clinical endpoint to determine the disease remission after active periodontal treatment and the actual benefit delivered to the patients [66]. Since residual pockets with bleeding on probing are strong indicators for inflammation and periodontal disease recurrence [67], thus these parameters were used in the present trial as clinical endpoints to reflect the patient's clinical health. Accordingly, treated sites reaching PD of 4 mm or less with no bleeding on probing were considered a reasonable cutoff point for reaching disease stability [68], which was equally achieved after treating the intraosseous defects of the stage III periodontitis patients with either PRF or CM with DFDBA.

However, it is important to interpret the present findings in the context of the limitations of the current study. First, the recently developed PRF horizontal centrifugation/preparation process [15] was not used in the current study. This method could have further boosted the number of leukocytes and platelets within the PRF membrane, with platelets more evenly distributed. This advancement should be considered in future trials. Second, due to the nature of the procedure, blinding of participants was not feasible in the test group, requiring blood sample collection. Third, the calibration of the outcome assessors should have been conducted on a higher number of patients. Fourth, because the employment of autogenous blood-originating products relies on patients' approval, participants who feared blood collection declined to take part in the trial. Fifth, despite the fact that minimally invasive surgical techniques (MIST) remain presently advocated in regenerative treatments of intraosseous defects [42], such techniques were not feasible in the present trial's design. A standard OFD was employed since the included morphologically challenging noncontained defects with loss of buccal and lingual walls demanded more mucoperiosteal flap extension for proper defect accessibility and DFDBA and barrier membrane application [5].

Within the present study's limitations, it can be inferred that both treatments (low-speed PRF + DFDBA and CM + DFDBA) significantly enhanced all clinical and radiological parameters 12 months postsurgically. PRF membranes could thus be carefully considered an alternative option for a barrier membrane in combination with allogenic bone grafts, even in intraosseous periodontal defects with challenging morphology. Finally, further histological and clinical investigations with larger sample sizes are required to investigate the regenerative aptitude of low-speed PRF membranes in combination with various bone grafting materials in the surgical treatment of intraosseous periodontal defects.

## Figures and Tables

**Table 1:** Baseline characteristics.

Parameter	Low speed-PRF + DFDBA (*n* = 11)	CM + DFDBA (*n* = 11)	*p*-Value
Age (years, mean ± SD)	32.64 ± 6.82	34.55 ± 8.31	0.562
Gender (*n* [%])			
Male	6 (54.5%)	2 (18.2%)	0.076
Female	5 (45.5%)	9 (81.8%)	—
Tooth location (*n* [%])			
Anterior	5 (45.5%)	3 (27.3%)	0.375
Posterior	6 (54.5%)	8 (72.7%)	—
Intrabony defect morphology (*n* [%])			
1 wall	4 (36.4%)	5 (45.5%)	0.665
Combined 1–2 walls	7 (63.6%)	6 (54.5%)	—
Radiographic defect angle (degree)			
Baseline	41.26 ± 18.40	36.34 ± 7.67	0.423

*Note:* Age comparison with independent *t*-test, gender, and periodontal defect characteristics comparison with chi-square test, radiographic defect angle with independent *t*-test for intergroup comparison.

Abbreviations: CM, collagen membrane; DFDBA, demineralized freeze-dried bone allograft; PRF, platelets-rich fibrin.

**Table 2:** Clinical outcomes.

Parameter	Low speed-PRF + DFDBA Mean (±SD)	CM + DFDBA Mean (±SD)	Intergroup *p*-Value
CAL (mm)			
Baseline	7.18 ± 2.64	6.73 ± 1.56	0.628
3 m	4.73 ± 2.00	3.91 ± 1.45	0.285
6 m	4.27 ± 1.62	3.45 ± 0.93	0.162
9 m	4.27 ± 1.19	3.73 ± 1.00	0.260
12 m	4.36 ± 1.21	4.09 ± 1.14	0.591
Intragroup *p*-value	<0.001*⁣*^*∗*^	<0.001*⁣*^*∗*^	—
mm gain (3 m)	2.45 ± 1.51	2.82 ± 1.25	0.545
% gain (3 m)	33.55 ± 19.10	42.15 ± 15.89	0.265
mm gain (6 m)	2.91 ± 1.70	3.27 ± 1.27	0.576
% gain (6 m)	39.57 ± 14.61	47.95 ± 11.49	0.150
mm gain (9 m)	2.91 ± 1.87	3.00 ± 1.41	0.899
% gain (9 m)	39.57 ± 16.06	43.54 ± 15.65	0.400
mm gain (12 m)	2.82 ± 1.83	2.64 ± 1.50	0.802
% gain (12 m)	37.40 ± 10.49	37.93 ± 17.35	0.932

**PD (mm)**	**Median (25, 75 percentile)**	**Median (25, 75 percentile)**	

Baseline	6.00 (6.00, 6.00)	6.00 (6.00, 7.00)	0.750
3 m	3.00 (3.00, 4.00)	3.00 (3.00, 4.00)	0.972
6 m	3.00 (3.00, 4.00)	3.00 (3.00, 3.00)	0.246
9 m	3.00 (3.00, 4.00)	3.00 (3.00, 4.00)	0.484
12 m	4.00 (3.00, 4.00)	4.00 (3.00, 4.00)	0.655
Intragroup *p*-value	<0.001*⁣*^*∗*^	<0.001*⁣*^*∗*^	—
mm reduction 3 m	3.00 (2.00, 4.00)	3.00 (2.00, 3.00)	1.00
% reduction 3 m	50.00 (33.33, 57.14)	50.00 (40.00, 50.00)	0.840
mm reduction 6 m	3.00 (2.00, 4.00)	3.00 (3.00, 4.00)	0.384
% reduction 6 m	50.00 (33.33, 57.14)	50.00 (50.00, 57.14)	0.333
mm reduction 9 m	3.00 (2.00, 3.00)	3.00 (2.00, 4.00)	0.488
% reduction 9 m	50.00 (33.33, 50.00)	50.00 (40.00, 57.14)	0.451
mm reduction 12 m	2.00 (2.00, 3.00)	2.00 (2.00, 3.00)	0.914
% reduction 12 m	40.00 (33.33, 50.00)	42.86 (33.33, 50.00)	0.891
Intragroup *p*-value	0.514	0.006*⁣*^*∗*^	—

**GRD (mm)**	**Median (25, 75 percentile)**	**Median (25, 75 percentile)**	

Baseline	0.00 (0.00, 1.00)	0.00 (0.00, 1.00)	0.745
Recession at 3 m	1.00 (0.00, 2.00)	1.00 (0.00, 1.00)	0.131
Recession at 6 m	1.00 (0.00, 1.00)	0.00 (0.00, 1.00)	0.185
Recession at 9 m	1.00 (0.00, 1.00)	1.00 (0.00, 1.00)	0.401
Recession at 12 m	1.00 (0.00, 1.00)	1.00 (0.00, 1.00)	0.802
Intragroup *p*-value	0.070	0.311	—
FMPS (%)			
Baseline (%)	16.45 ± 2.07	18.00 ± 2.15	0.101
12 months (%)	11.00 ± 1.18	11.64 ± 1.12	0.210
Intragroup *p*-value	<0.001*⁣*^*∗*^	<0.001*⁣*^*∗*^	—
FMBS (%)			
Baseline (%)	17.91 ± 1.04	17.64 ± 2.69	0.757
12 months (%)	10.91 ± 0.54	10.55 ± 0.82	0.233
Intragroup *p*-value	<0.001*⁣*^*∗*^	<0.001*⁣*^*∗*^	—

*Note:* CAL comparison with independent *t*-test for intergroup comparison and repeated measure ANOVA for intragroup comparison, PD and GRD comparison with independent *t*-test for intergroup comparison and paired *t*-test for intragroup comparison, FMPS and FMBS comparison with independent *t*-test for intergroup comparison and repeated measure ANOVA for intragroup comparison.

Abbreviations: CAL, clinical attachment level; CM, collagen membrane; DFDBA, demineralized freeze-dried bone allograft; FMBS, full mouth bleeding score; FMPS, full mouth plaque score; GRD, gingival recession depth; PD, probing depth; PRF, platelets -rich fibrin.

*⁣* ^*∗*^Statistically significant at *p* < 0.05.

**Table 3:** Descriptive statistics and results of repeated measures ANOVA test for comparison between PD measurements (mm) in the two groups and the changes within each group at the clinical endpoint.

Time	Low speed PRF + DFDBA (*n* = 11)	CM + DFDBA (*n* = 11)	*p*-Value	Effect size (partial eta squared)
	Mean (± SD)	Mean (± SD)		
Baseline	6.36 ± 1.29	6.36 ± 1.03	1	0
12 months	3.64 ± 0.5	3.73 ± 0.47	0.666	0.01
*p*-Value	<0.001*⁣*^*∗*^	<0.001*⁣*^*∗*^	—	—
Effect size (partial eta squared)	0.768	0.756	—	—

Abbreviations: CM, collagen membrane; DFDBA, demineralized freeze-dried bone allograft; PD, probing depth; PRF, platelets-rich fibrin.

*⁣* ^*∗*^Significant at *p* < 0.05.

**Table 4:** Radiographic outcomes.

Parameter	Low speed PRF + DFDBA (*n* = 11)	CM + DFDBA (*n* = 11)	*p*-Value
RLDD (mm)			
Baseline	6.71 ± 1.35	6.77 ± 0.98	0.900
6 m	5.25 ± 1.18	5.03 ± 0.86	0.625
9 m	3.88 ± 0.90	4.27 ± 1.07	0.364
12 m	2.79 ± 0.79	3.01 ± 0.86	0.544
Intragroup *p*-value	<0.001*⁣*^*∗*^	<0.001*⁣*^*∗*^	—
Radiographic bone fill (mm)			
6 m	1.46 ± 0.39	1.75 ± 0.47	0.139
9 m	2.83 ± 0.71	2.50 ± 0.76	0.309
12 m	3.92 ± 0.90	3.76 ± 0.51	0.625
Intragroup *p*-value	<0.001*⁣*^*∗*^	<0.001*⁣*^*∗*^	—
Radiographic bone fill (%)			
6 m	21.95 ± 5.34	25.77 ± 6.31	0.141
9 m	42.06 ± 5.79	37.06 ± 9.65	0.156
12 m	58.40 ± 7.41	56.06 ± 7.23	0.464
Intragroup *p*-value	<0.001*⁣*^*∗*^	<0.001*⁣*^*∗*^	—

*Note:* RLDD and Radiographic bone fill comparison with Independent *t*-test for intergroup comparison and repeated measure ANOVA for intragroup comparison.

Abbreviations: CM, collagen membrane; DFDBA, demineralized freeze-dried bone allograft; PRF, platelets-rich fibrin; RLDD, radiographic linear defect depth.

*⁣* ^*∗*^Statistically significant *p* < 0.05.

**Table 5:** Results of linear regression analysis model showing predictors of CAL after 12 months.

Variables	Unstandardized coefficients		*p*-Value	95% Confidence Interval for *B*	
	*B*	SE		Lower limit	Upper limit
Site	−1.607	0.38	<0.001*⁣*^*∗*^	−2.4	−0.814

Abbreviations: CAL, clinical attachment level; SE, Standard Error.

*⁣* ^*∗*^Significant at *p* < 0.05.

## Data Availability

All data are available from the corresponding author upon reasonable request.

## Nomenclature

BMPs: Bone morphogenetic proteins
CAL: Clinical attachment level
CMs: Collagen membranes
DFDBA: Demineralized freeze-dried bone allograft
FGF-*β*: Fibroblast growth factor-*β*
FMBS: Full mouth bleeding score
FMPS: Full mouth plaque score
GRD: Gingival recession depth
GTR: Guided tissue regeneration
IGF: Insulin-like growth factor
MIST: Minimally invasive surgical techniques
PD: Probing depth
PDGF: Platelet-derived growth factor
PRF: Platelet-rich fibrin
RLDD: Radiographic bone fill and radiographic linear defect depth
TGF-*β*: Transforming growth factor-*β*
VEGF: Vascular endothelial growth factor.
